# Factors associated with two different stroke mechanisms in perforator infarctions regarding the shape of arteries

**DOI:** 10.1038/s41598-022-21329-7

**Published:** 2022-10-06

**Authors:** Sang Hee Ha, Jae-Chan Ryu, Jae-Han Bae, Sujin Koo, Jun Young Chang, Dong-Wha Kang, Sun U. Kwon, Jong S. Kim, Dae-il Chang, Bum Joon Kim

**Affiliations:** 1 Department of Medicine, Graduate School, Kyung Hee University, Seoul, South Korea; 2 Department of Neurology, Asan Medical Center, University of Ulsan College of Medicine, 388-1 Pungnap-Dong, Songpa-Gu, Seoul, 138-736 South Korea; 3 Graduate School of Converging Clinical and Public Health, Ewha Woman’s University, Seoul, South Korea; 4 Department of Neurology, Kyung Hee University Hospital, Seoul, South Korea; 5 Department of Neurology, Gangneung Asan Hospital, Gangneung, South Korea

**Keywords:** Anatomy, Neurology

## Abstract

Two different stroke mechanisms are involved in small vessel disease: branch atheromatous disease (BAD) and lipohyalinotic degeneration (LD). We compared mechanisms of stroke in lenticulostriate arteries (LSA) vs. anterior pontine arteries (APA) and verified factors associated with stroke mechanisms, including shape of middle cerebral artery (MCA) and basilar artery (BA). We retrospectively reviewed patients with acute ischemic stroke with penetrating artery territory confirmed by MRI. The mechanisms of stroke were categorized based on diffusion-weighted imaging; BAD was defined as lesion larger than 10 mm in LSA and lesions involving basal pontine in APA. Other lesions were classified as LD. The shapes of MCA and BA were classified as straight, with one angle, or with two angles (U, C or S shape, respectively) using anterior–posterior view. The study included 221 patients. LD was more common in LSA infarcts, but BAD was more common in APA infarcts (p < 0.001). Low initial National Institutes of Health Stroke Scale [Adjusted Odds ratio (aOR) = 0.78; p < 0.001], absence of hyperlipidemia [aOR = 0.31; p = 0.002], previous statin use [aOR = 4.35; p = 0.028] LSA infarcts [reference = APA territory; aOR = 11.07; p < 0.001], and S-shaped vessels (reference = straight shaped vessels; aOR = 3.51; p = 0.004) were independently associated with LD. Angulations in the mother vessels may be more associated with true small vessel disease more with LD than BAD.

## Introduction

Cerebral small vessel occlusive disease (CSVD) is characterized by a diseased perforating artery in the brain, which leads to ischemic damage^1^. The typical perforating arteries arise from the middle cerebral artery (MCA), including the lenticulostriate arteries (LSA), and from the basilar artery (BA), including the anterior pontine arteries (APA). Previous studies demonstrated several differences in risk factors for stroke between infarctions of anterior and posterior circulation^2^.

Risk factors for stroke also differ according to the mechanisms of stroke^3^. Perforating arteries are involved in infarctions by two major different mechanisms: branch atheromatous disease (BAD) and lipohyalinotic degeneration (LD)^4^. BAD is presumably related to an atheroma of the mother artery obliterating the orifice of the perforating arteries and is radiologically defined as lesion larger than 10 mm in LSA and lesions involving basal pontine in APA. LD is associated with damage of the perforating artery itself^5^. BAD is more frequently observed among APA infarctions compared with LSA infarctions^3^. The majority of APA infarction show basilar artery plaques in high resolution magnetic resonance images (MRI)^6^.

Arterial tortuosity, which was associated with hereditary condition, advanced age, and hypertension^7^, can induce hemodynamic alteration and trigger the development of atherosclerosis in a specific vessel, leading to BAD^8^. Also, it was known to be closely associated with LD^1^. Despite the potential importance of arterial tortuosity as a factor that may influence the stroke mechanism in perforating artery territories, the relationship between the arterial tortuosity and stroke mechanism has not been clearly verified. Therefore, the aim of the present study is to compare the vascular risk factors and imaging characteristics of LSA and APA infarcts between BAD and LD and evaluate whether the shape of the MCA and BA are associated with the mechanisms of stroke.

## Results

During the study period, 1592 patients were admitted to our stroke center and 320 (20.1%) patients were diagnosed as acute ischemic stroke due to small vessel occlusion. Finally, 221 (13.8%) patients satisfied the inclusion criteria. Among them, 136 (61.5%) patients had LSA infarcts and 85 (38.5%) had APA infarcts; 106 (48.0%) patients were classified as BAD and 115 (52.0%) patients were classified as LD. Regarding the shape of MCA or BA, 80 (36.2%) patients had straight MCA or BA, 69 (31.2%) patients had U- or C-shaped vessels, and 72 (32.6%) patients had S-shaped vessels. The inter-rater reliability for assessing the shape of vessel was good (Cronbach's Alpha = 0.817).

### Lenticulostriate artery vs. anterior pontine artery infarcts

The baseline characteristics of the patients are shown in Table 1. There was no significant difference in demographics and risk factors between the two groups, except diabetes mellitus was less frequent in LSA than in the APA infarcts (26.5 vs. 40.0%, respectively; p = 0.035). The mechanism of stroke differed according to the location; LD was more frequent in LSA infarctions, whereas BAD was more common in the APA infarctions (p < 0.001). The degree of stenosis differed between the two groups; no stenosis was more common in LSA infarctions, whereas stenosis of less than 50% were more common in APA infarctions (p < 0.001). The shape of MCA and BA differed between the LSA and APA infarctions (p < 0.001); patients with APA infarctions had more straight and U- or C-shaped vessels, whereas patients with LSA infarctions had more S-shaped vessels (p < 0.001; Table 1). There was no difference of the presence of posterior communication arteries among these groups.

**Table 1:** Baseline characteristics in patients with LSA and APA infarctions.

	LSA (n = 136)	APA (n = 85)	*P*-value
Age (years)	66 ± 12	67 ± 10	0.700
Male	66 (48.5)	49 (57.6)	0.187
Hypertension	104 (76.5)	62 (72.9)	0.555
Diabetes mellitus	36 (26.5)	34 (40.0)	0.035
Hyperlipidemia	71 (52.2)	42 (49.4)	0.686
Smoking history	44 (32.4)	38 (44.7)	0.064
Previous stroke history	34 (25.0)	23 (27.1)	0.734
Previous antiplatelet	17 (12.5)	10 (11.8)	0.871
Previous statin	15 (11.0)	8 (9.4)	0.702
Initial NIHSS (IQR)	3 (1–5)	4 (1–5)	0.861
**Stroke mechanism**			< 0.001
BAD	37 (27.2)	69 (81.2)	
LD	99 (72.8)	16 (18.8)	
**White matter hyperintensities**			0.496
0	18 (13.2)	10 (11.8)	
1	68 (50.0)	50 (58.8)	
2	35 (25.7)	15 (17.6)	
3	15 (11)	10 (11.8)	
Microbleeds	42 (30.9)	26 (30.6)	0.963
Lacunes	58 (42.6)	32 (37.6)	0.462
Presence of Pcom	39 (28.7)	22 (25.9)	0.651
**Stenosis degree**			0.001
No stenosis	97 (71.3)	41 (48.2)	
< 50% stenosis	39 (28.7)	44 (51.8)	
**MCA or BA shape**			< 0.001
Straight	46 (33.8)	34 (40)	
U or C shape	27 (19.9)	42 (49.4)	
S shape	63 (46.3)	9 (10.6)	

Results presented as number (%) or mean ± SD or IQR.

LSA: lenticulostriate arteries, APA: anterior pontine arteries, MCA: middle cerebral artery, BA: basilar artery, BAD: branch atheromatous disease, LD: lipohyalinotic degeneration; NIHSS: National Institutes of Health Stroke Scale.

### Branch atheromatous disease vs. lipohyalinotic disease in each territory

Of the 136 patients with LSA territory infarctions, 37 (27.2%) patients had BAD and the remaining 99 (72.8%) had LD. Hyperlipidemia was more frequently observed in patients with BAD than those with LD (70.3 vs. 45.5%, respectively, p = 0.01). No other significant differences in risk factors were detected between patients with BAD and LD. The initial National Institutes of Health Stroke Scale (NIHSS) scores were higher in patients with BAD than those with LD [4 (2–6) vs. 3 (1–5), respectively; p = 0.035]. The White matter hyperintensities (WMHs) were less severe in patients with BAD than those with LD (p = 0.014; Table 2). The shape of the MCA differed according to the mechanisms of stroke (*p* = 0.026). The straight (37.8%) was more prevalent in BAD, and the S shaped (52.5%) was more frequently observed in patients with LD.

**Table 2:** Characteristics in the BAD and LD groups in the two different territories.

	LSA		*P*-value	APA		*P*-value
	BAD (n = 37)	LD (n = 99)		BAD (n = 69)	LD (n = 16)	
Age (years)	64 ± 13	67 ± 11	0.152	68 ± 11	63 ± 8	0.051
Male	17 (45.9)	49 (49.5)	0.712	40 (58)	9 (56.3)	0.9
Hypertension	26 (70.3)	78 (78.8)	0.297	54 (78.3)	8 (50)	0.022
Diabetes mellitus	12 (32.4)	24 (24.2)	0.335	29 (42)	5 (31.3)	0.428
Hyperlipidemia	26 (70.3)	45 (45.5)	0.01	35 (50.7)	7 (43.8)	0.615
Smoking history	15 (40.5)	29 (29.3)	0.212	28 (40.6)	10 (62.5)	0.112
Previous stroke history	9 (24.3)	25 (25.3)	0.911	18 (26.1)	5 (31.3)	0.675
Previous antiplatelet	2 (5.4)	15 (15.2)	0.126	10 (14.5)	0	0.105
Previous statin	1 (2.7)	14 (14.1)	0.058	6 (8.7)	2 (12.5)	0.639
Initial NIHSS (IQR)	4 (2–6)	3 (1–5)	0.035	4 (2–5)	1 (0–3)	0.001
**White matter hyperintensities**			0.014			0.186
0	10 (27)	8 (8.1)		6 (8.7)	4 (25)	
1	19 (51.4)	49 (49.5)		43 (62.3)	7 (43.8)	
2	6 (16.2)	29 (29.3)		13 (18.8)	2 (12.5)	
3	2 (5.4)	13 (13.1)		7 (10.1)	3 (18.8)	
Microbleeds	7 (18.9)	35 (35.4)	0.065	20 (29)	6 (37.5)	0.505
Lacunes	11 (29.7)	47 (47.5)	0.063	59 (37.7)	6 (37.5)	0.989
**Stenosis degree**			0.795			0.017
No stenosis	27 (73)	70 (70.7)		29 (40.6)	12 (68.8)	
< 50% stenosis	10 (27)	29 (29.3)		40 (59.4)	4 (31.3)	
**MCA or BA shape**			0.026			0.281
Straight	14 (37.8)	32 (32.3)		30 (43.5)	4(25)	
U or C shape	12 (32.4)	15 (15.2)		33 (47.8)	9 (56.3)	
S shape	11 (29.7)	52 (52.5)		6 (8.7)	3 (18.8)	

Results presented as number and percent (%) or mean ± SD or IQR.

LSA: Lenticulostriate arteries, APA: Anterior pontine arteries, MCA: middle cerebral artery, BA: basilar artery, BAD: branch atheromatous disease, LD: lipohyalinotic degeneration; NIHSS: National Institutes of Health Stroke Scale.

Among the 85 patients with APA infarctions, 69 (81.2%) patients had BAD, and 16 (18.8%) patients had LD. The prevalence of hypertension was higher in patients with BAD than those with LD (78.3 vs. 50%, respectively; p = 0.022). The initial NIHSS scores were higher in patients with BAD than those with LD [4 (2–5) vs. 1 (0–3), respectively; p = 0.001]. No significant differences in BA shapes were detected between BAD and LD in patients with APA infarctions (p = 0.281; Table 2).

### Factors associated with LD

Diabetes mellitus [Odds ratio (OR) = 0.54, 95% confidence interval (CI) 0.301–0.949; p = 0.033], initial NIHSS score (OR = 0.85, 95% CI 0.761–0.941; p = 0.002), LSA infarction (reference = APA infarction. OR = 11.54, 95% CI 5.951–22.372; p < 0.001), stenosis less than 50% (OR = 0.45 95% CI 0.259–0.786; p = 0.005), white matter hyperintensities (reference: Fazekas scale 0–1, OR = 1.93 95%CI 1.089–3.404; p = 0.024), and S-shaped vessels (reference: straight vessel, OR = 3.95 95% CI 1.964–7.962); p < 0.001) were independently associated with LD.

In the multivariable analysis, absence of hyperlipidemia (OR = 0.31 95% CI 0.134–0.651; p = 0.002), previous statin use (OR = 4.35 95%CI 1.175–16.114; p = 0.028), low initial NIHSS (OR = 0.78 95% CI 0.679–0.895; p < 0.001), LSA territory [reference = APA territory, OR = 11.07 95% CI 5.007–24.470; p < 0.001] and S-shaped vessels (reference = straight vessel, OR = 3.51. 95% CI 1.486–8.265; p = 0.004) were independently associated with LD (Table 3). Especially in the subgroup analysis with patients with LSA infarction, S-shaped vessel (reference = Others, OR = 3.98. 95% CI 1.551–10.194; p = 0.004) was associated with LD (Supplementary Table 1).

**Table 3:** Factors associated with LD.

	Unadjusted univariate analysis		Adjusted multivariate analysis^a^	
	OR (95% CI)	*P*-value	OR (95% CI)	*P*-value
Age (years)	1.00 (0.977–1.022)	0.977	-	
Male	0.88 (0.516–1.484)	0.620	–	
Hypertension	0.94 (0.523–1.775)	0.906		
Diabetes mellitus	0.54 (0.301–0.949)	0.033		
Hyperlipidemia	0.61 (0.358–1.037)	0.068	0.31 (0.143–0.651)	0.002
Smoking history	0.75 (0.435–1.299)	0.307		
Previous stroke history	1.03 (0.565–1.888)	0.917		
Previous antiplatelet	1.18 (0.523–2.640)	0.696		
Previous statin	2.29 (0.901–5.798)	0.082	4.35 (1.175–16.114)	0.028
Initial NIHSS	0.85 (0.761–0.941)	0.002	0.78 (0.679–0.895)	< 0.001
**White matter hyperintensities**				
0–1	1 (reference)		1 (reference)	
2–3	1.93 (1.089–3.404)	0.024	2.06 (0.960–4.406)	0.064
Microbleeds	1.62 (0.908–2.896)	0.103		
Lacunes	1.59 (0.927–2.741)	0.092		
**Location**				
APA	1 (reference)		1 (reference)	
LSA	11.54 (5.951–22.372)	< 0.001	11.07 (5.007–24.470)	< 0.001
**Stenosis degree**				
No stenosis	1 (reference)			
< 50% stenosis	0.45 (0.259–0.786)	0.005		
**MCA or BA shape**				
Straight	1 (reference)		1 (reference)	
U or C shape	0.65 (0.336–1.265)	0.206	0.86 (0.360–2.035)	0.726
S shape	3.51 (1.486–8.265)	0.004	3.51 (1.486–8.265)	0.004

Results are presented by OR (95% CI).

LSA: Lenticulostriate arteries, APA: Anterior pontine arteries, MCA: middle cerebral artery, BA: basilar artery, LD: lipohyalinotic degeneration; NIHSS: National Institutes of Health Stroke Scale.

^a^Multivariate logistic regression adjusted for age, sex, initial NIHSS, Diabetes mellitus, Hyperlipidemia, Previous statin, location, stenosis degree and MCA or BA shape.

## Discussion

In the present study, LD was more frequent in LSA infarctions, whereas BAD was more frequent in APA infarctions. Regarding the artery shapes, the S-shaped was the most frequent in the MCA and the C-shaped was the most common in the BA. Among those with LSA infarctions, patients with LD showed more severe WMHs lesions and more S-shaped MCA compared with BAD patients. Among patients with APA infarctions, BAD patients showed more hypertension and more frequent stenosis in the BA. Finally, from the multivariable analysis, LSA infarction and S-shaped artery were independently associated with LD.

The tortuosity of intracranial arteries is associated with small vessel disease, including lacunar infarctions. While the reason for this association is unclear, several hypotheses were proposed: the distortion and obstruction of the perforating arteries arising from the tortuous intracranial arteries may result in ischemic damage to the perforator territories^7^. The hemodynamic stress stemming from the high tortuosity may lead to endothelial dysfunction and direct injuries to the perforators^9^. Second, the tortuous intracranial arteries may develop an area of blood flow with turbulence (eddying flow) and blood flow to the perforators originating from that particular area may be reduced, causing ischemia^8,10^. Finally, higher tortuosity was well known to be associated with higher grade WMH^11^ and previous studies showed increased arterial stiffness was associated with white matter lesions^12^. There have been few reports that arterial stiffness increased in patients with tortuous vessels^13^, but the relationship between intracranial tortuosity and atrial stiffness is still unknown. Further study should be needed to evaluate our results.

We previously showed that the shape of intracranial arteries was associated with the location and presence of atherosclerotic plaques inside the intracranial arteries^8,14^. These findings were explained by the low shear stress in the inner curvature of the tortuous vessel, enhancing the development and progression of atheroma in the intracranial arteries^8^. However, the current study with patients with small vessel disease showed that vessels with more angulation were associated with LD rather than BAD. Based on our current results, the association between tortuous arteries and small vessel disease may not be due to the development of atherosclerosis in the vessel wall. The shape of the vessels may alter hemodynamics leading to atherosclerosis, but also can affect the perforators branching from the tortuous artery and cause lipohyalinotic small vessel disease. The prevalence of LD was especially high in those with S-shaped artery and S-shape artery was independently associated with LD, but not the U or C-shaped arteries. A rapidly alternating flow in a S-shaped vessel maybe critical to induce the turbulence flow or to exceed the threshold leading to direct injury of the perforators.

Differences in the shape of the MCA and BA may at least partially explain the differences in the stroke mechanisms between LSA and APA infarctions. Patients with LSA infarctions exhibited more LD than patients with APA infarctions. The most frequent vessel shape in the MCA was the S-shaped with two angles, whereas the most frequent shape in the BA was C-shaped with a single angulation. Diabetes is also a known risk factor for infarctions in the posterior circulation^2^. Metabolic syndrome, including diabetes, is also a major risk factor for intracranial atherosclerosis^15^. The high prevalence of diabetes in patients with APA infarctions may explain the higher prevalence of BAD in APA infarctions compared with the prevalence in LSA infarctions. However, when we compared LD and BAD among APA territory infarctions, diabetes was not related to the stroke mechanism and only hypertension was significantly different between the two groups. The prevalence of diabetes was higher in APA infarctions than LSA infarctions, regardless of the stroke mechanism. Based on these findings, diabetes may affect both types of stroke mechanisms in the posterior circulation, which is more vulnerable to injuries stemming from sympathetic denervation and impaired vascular tone by diabetic neuropathies than the anterior circulation^16^. High blood pressure, which was a significant risk factor associated with BAD in APA infarctions, may augment the development of atherosclerosis in BA in patients with BAD^17^. Pons are supplied by various types of perforators (paramedian, short and long circumflex arteries), and the collaterals are more developed than in the LSA territory. Because of the better collaterals, LD in a single perforator may less cause APA infarction whereas, BAD obliterating various types of perforators simultaneously may be more important in the occurrence of APA infarctions^18^.

Our study has several limitations. First, the number of strokes was small and the study was performed in a single center. Second, the diagnosis of BAD in the present study was defined using conventional MRI. Discriminating BAD from LD was not always clear. However, there was a significant difference in the stenosis and imaging biomarkers of small vessel disease between BAD and LD. The trend of BAD and LD in LSA and APA stroke was also similar to the previous study^19^. A study with high resolution MRI may be helpful in distinguishing BAD from LD in the future. Third, the tortuosity was not quantitatively measured. Therefore, it was hard to accurately distinguish the difference between the sharp C-shaped and the gentle C-shaped. However, classifying the shape of MCA and BA was intuitive and the inter-rater reliability was good. Finally, data describing the normal variation of the shape of MCA and BA is scanty. Therefore, it is difficult to say that the shape of intracranial artery is associated with the occurrence of stroke.

However, our data shows that S-shaped MCA with more angles and high tortuosity is associated with LD rather than BAD. As the strategy for secondary stroke prevention differ according to the stroke mechanism, understanding and diagnosing the exact mechanism of stroke may be important. The differences in vessel shape between MCA and BA may help explain the higher prevalence of LD in LSA infarctions and BAD in APA infarctions.

## Methods

### Participants

We retrospectively reviewed patients with acute (< 7 days from stroke onset) penetrating artery territory infarctions confirmed by MRI who were admitted to the Asan Medical Center, Seoul, South Korea from May 2019 to December 2020. Patients were included in this study if they had small infarcts no greater than 20 mm in diameter located within the territories of the LSA or APA^19^ and had minimal to mild stenosis (< 50%) or no stenosis shown on magnetic resonance angiography (MRA). Lesions involved the thalamus and other brain stem lesions, such as medulla and midbrain, were excluded because geometrical properties could not be assessed properly.

We excluded patients who had any of the following: (1) a significant (> 50%) stenosis of the corresponding extracranial or intracranial artery; (2) infarcts located at the cortex or border zone or acute multiple infarctions; (3) any potential causes of embolisms (i.e., embolic heart disease or coagulopathy); (4) other causes of intracranial stenosis regardless of the degree of stenosis (i.e., Moyamoya disease or intracranial arterial dissection); or (5) other genetic disease associated with small vessel diseases (i.e., Cerebral autosomal dominant arteriopathy with subcortical infarcts and leukoencephalopathy (CADASIL) or Fabry disease). The local ethics committee, ASAN medical center, South Korea, approved this study (IRB number: S2021-1879-0001) and informed consent was waived due to retrospective nature of the study. All methods of this study were performed following the relevant guidelines and regulations.

### Clinical and imaging characteristics

Demographic data and risk factors were obtained by reviewing medical records and stroke registry database records. The presence of risk factors, comorbidities, and medication use on stroke onset were assessed. The neurological deficit of stroke was evaluated using the NIHSS score at admission.

**Figure 1:**
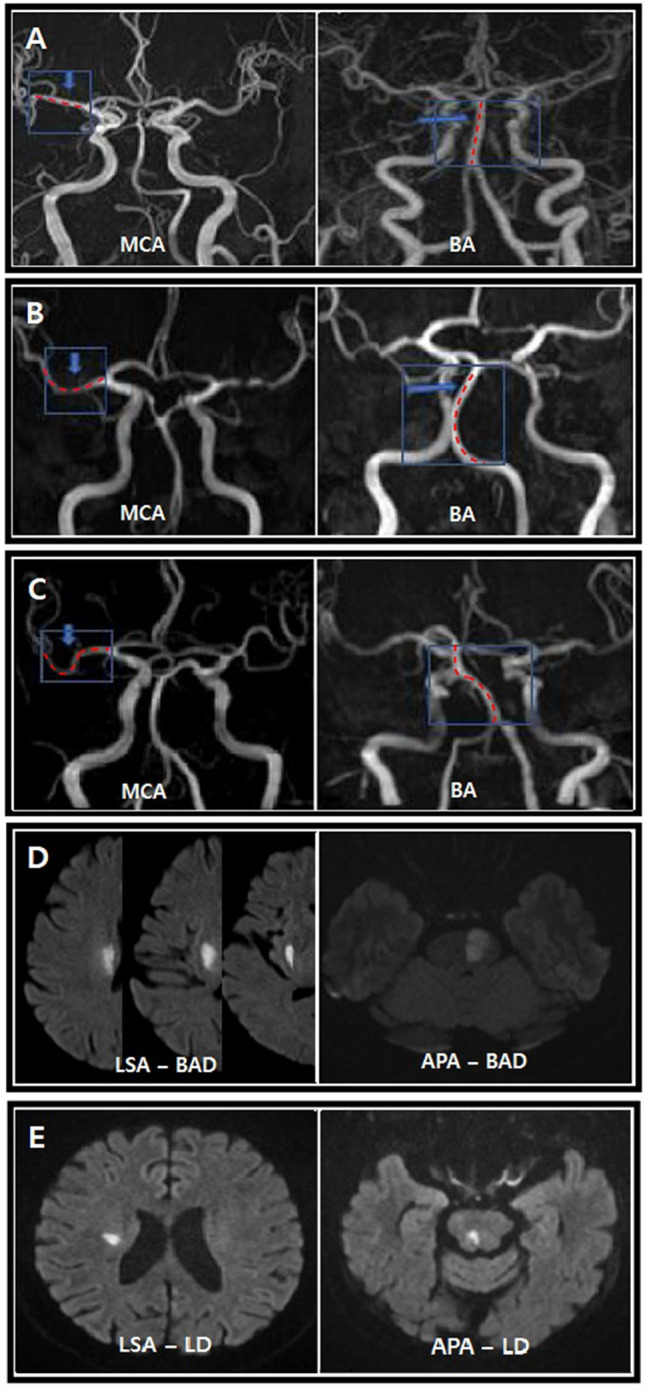
Shape of MCA or BA and BAD and LD in each territory; Straight (**A**), U-shaped or C-shaped (**B**), and S-shaped (**C**); BAD (**D**) and LD (**E**). Branch atheromatous disease, LD; Lipohyalinotic degeneration LSA; lenticulostriate artery, APA; Anterior pontine artery; MCA: middle cerebral artery; BA: basilar artery.

Stenosis of the intracranial artery was evaluated based on the time-of-flight (TOF) MRA from the MCA and BA, using the anterior–posterior view. The shapes of symptomatic vessels were evaluated. For the MCA, two lines starting from ACA-MCA bifurcation point and MCA bifurcation point and running through the midline of MCA were drawn. For BA, standard line length was drawn from the top of the BA to the union of both vertebral arteries. Then we counted the angulation number. Based on these, the ipsilesional MCA and BA shapes were classified into three groups: 1) straight; 2) with a quadratic curve (single angulation; U shape for MCA and C shape for BA); and 3) a cubic curve (double angulation; S shape for MCA or BA) (Fig. 1A–C)^8,20^. The severity of stenosis was divided into two categories: mild stenosis was defined as signal reduction to less than 50% of the nearest normal sized vessel and no stenosis was defined as a normal looking vessel^21^.

Small vessel burden was evaluated using fluid-attenuated inversion recovery (FLAIR) image. WMHs were defined as periventricular lesions appearing hyperintense on the T2-weighted FLAIR images. WMHs were grouped into four grades according to the modified Fazekas scale (0 = absent; 1 = pencil-thin lining; 2 = halo of ≥ 5 mm thickness; 3 = irregular white matter hyperintensities extending into the deep white matter). Cerebral microbleeds (CMB) were identified as small perivascular hemosiderin deposits, which could be visualized as small, rounded, homogeneous, and hypointense lesions on T2*-weighed gradient-recalled echo or susceptibility-weighted MRI. For this study, all CMBs located deep and lobar were included. Lacunes were defined as chronic small cavities that presumably represent the healed stage of a lacunar infarct^5,11^.

### Stroke mechanisms detected by MRI

BAD was defined to correspond with infarcts caused by occlusion of the orifices or proximal portions of penetrating arteries. Also, based on the diffusion-weighted imaging, BAD of the LSA was defined as infarcts more than 10 mm in diameter on the axial slice and visible for 4 or more axial slices at a slice thickness of 7 mm. BAD of the APA was defined as unilateral infarcts extending to the basal surface of the pons^19,22^.

Other infarcts that were not classified as BAD in either the LSA or APA were classified as LD. However, a few patients could not be clearly categorized as LD or BAD with the first MRI. In these patients, the MRI was repeated within 2 days and the final diagnosis was based on the second MRI (Fig. 1D,E)^19^.

### Statistical analysis

The characteristics of LSA and APA infarctions were compared. Then, the characteristics were compared between BAD and LD among patients with LSA and APA. Chi-squared or Fisher’s exact tests for categorical variables and Student *t* test for continuous variables were used. Univariable and multivariable analyses were used to investigate the factors associated with the stroke mechanisms (BAD vs. LD). We only included variables showing potential association (p < 0.1) in the multivariate logistic model. IBM SPSS version 21.0 software (SPSS, Chicago, IL) was used for all analyses, and p values < 0.05 were considered statistically significant.

### Ethical approval

The local ethics committee, ASAN medical center, South Korea, approved this study (IRB number: S2021-1879-0001).

### Informed consent

Due to retrospective nature of the study, need for informed consent was waived by Institutional Review Board of Asan medical center.

## Supplementary Information

Supplementary Table 1.

## Data Availability

The datasets generated during and/or analysed during the current study are available in the Open Science Framework repository, https://osf.io/we7a6/.
