# Supplementary material for: Factors associated with two different stroke mechanisms in perforator infarctions regarding the shape of arteries
Source: Sci Rep. 2022 Oct 6;12:16752. doi: 10.1038/s41598-022-21329-7 (PMC9537294; doi:10.1038/s41598-022-21329-7)
Supplement: Supplementary file 1 — Supplementary Table 1. [file 41598_2022_21329_MOESM1_ESM.docx]

**Supplementary table 1. Factors associated with LD in LSA and APA territories**

|  | **LSA territory** | | | | **APA territory** | | | |
| --- | --- | --- | --- | --- | --- | --- | --- | --- |
|  | Crude OR | | Adjusted multivariate analysis^*^ | | Crude OR |  | Adjusted multivariate analysis^†^ | |
|  | OR (95% CI) | *P* | OR (95% CI) | *P* | OR (95% CI) | *P* | OR (95% CI) | *P* |
| Age (years) | 1.03 (0.994 – 1.057) | 0.114 | - |  | 0.95 (0.900 – 1.003) | 0.066 | 0.94 (0.876 – 1.001) | 0.005 |
| Male | 1.15 (0.541 – 2.458) | 0.713 | - |  | 0.93 (0.311 – 2.793) | 0.900 | - |  |
| Hypertension | 1.57 (0.669 – 3.691) | 0.300 |  |  | 0.28 (0.089 – 0.864) | 0.027 | 0.23 (0.062 – 0.864) | 0.029 |
| Diabetes mellitus | 0.67 (0.291 – 1.525) | 0.337 |  |  | 0.63 (0.197 – 2.000) | 0.430 |  |  |
| Hyperlipidemia | 0.35 (0.157 – 0.791) | 0.011 | 0.25 (0.095 – 0.672) | 0.006 | 0.76 (0.253 – 2.258) | 0.616 |  |  |
| Smoking history | 0.61 (0.277 – 1.334) | 0.214 |  |  | 2.44 (0.796 – 7.483) | 0.119 |  |  |
| Previous stroke history | 1.05 (0.437 – 2.527) | 0.911 |  |  | 1.29 (0.393 – 4.216) | 0.676 |  |  |
| Previous antiplatelet | 3.13 (0.679 – 14.391) | 0.144 |  |  | NA |  |  |  |
| Previous statin | 5.93 (0.751 – 46.795) | 0.091 |  |  | 1.50 (0.274 – 8.226) | 0.641 |  |  |
| Initial NIHSS | 0.84 (0.742 – 0.962) | 0.011 | 0.82 (0.698 – 0.969) | 0.019 | 0.62 (0.454 – 0.856) | 0.004 | 0.61 (0.429 – 0.867) | 0.006 |
| **White matter hyperintensities** |  |  |  |  |  |  | - |  |
| 0 | 1 (reference) |  | 1 (reference) |  | 1 (reference) |  |  |  |
| 1 | 3.22 (1.106 – 9.398) | 0.032 | 3.74 (1.067 – 13.077) | 0.039 | 0.24 (0.055 – 1.090) | 0.065 |  |  |
| 2 | 6.04 (1.681 – 21.718) | 0.006 | 10.52 (2.355 – 46.995) | 0.002 | 0.23 (0.033 – 1.628) | 0.141 |  |  |
| 3 | 8.13 (1.405 – 46.998) | 0.019 | 9.29 (1.362 – 63.387) | 0.023 | 0.64 (0.101 – 4.097) | 0.640 |  |  |
| Microbleeds | 2.34 (0.934 – 5.882) | 0.070 |  |  | 1.47 (0.471 – 4.587) | 0.507 |  |  |
| Lacunes | 2.14 (0.952 – 4.792) | 0.066 |  |  | 0.99 (0.323 – 3.051) | 0.989 |  |  |
| **Stenosis degree** |  |  |  |  |  |  | **-** |  |
| No stenosis | 1 (reference) |  |  |  | 1 (reference) |  |  |  |
| < 50% stenosis | 1.12 (0.481- 2.604) | 0.795 |  |  | 0.24 (0.071 – 0.825) | 0.023 |  |  |
| **MCA shape** |  |  |  |  |  |  |  |  |
| Others | 1 (reference) |  |  |  | 1 (reference) |  |  |  |
| S shape | 2.62 (1.166 – 5.866) | 0.002 | 3.98 (1.551 – 10.194) | 0.004 | 2.42 (0.536 – 10.958) | 0.250 |  |  |

LSA: Lenticulostriate arteries, APA: Anterior pontine arteries, MCA: middle cerebral artery, BA: basilar artery, BAD: branch atheromatous disease, LD: lipohyalinotic degeneration; NIHSS: National Institutes of Health Stroke Scale

* Multivariate logistic regression adjusted for age, male, hyperlipidemia, initial NIHSS, WMH, and MCA shape

† Multivariate logistic regression adjusted for age, male, hypertension, initial NIHSS, WMH, stenosis degree
